# Holidays in the Sun and the Caribbean’s Forgotten Burden of Neglected Tropical Diseases

**DOI:** 10.1371/journal.pntd.0000239

**Published:** 2008-05-28

**Authors:** Peter J. Hotez

**Affiliations:** Sabin Vaccine Institute and Department of Microbiology, Immunology, and Tropical Medicine, George Washington University Medical Center, Washington, D.C., United States of America

Almost 40 million people live on the islands, islets, and cays that comprise the Caribbean [1]. This is one of the most tourism-dependent regions in the world, with approximately one-quarter of the workforce involved in a business that in some way caters to tourists [2]. According to the Caribbean Tourism Organization (CTO), an association of government and private sector agencies that disseminates data on the region, almost 22 million visitors come to the Caribbean annually, where they spend an estimated US$21.6 billion [3]. More than 80% of these visitors are people of means from the United States (11.4 million), Canada (1.7 million), and Europe (5.3 million), almost all of whom come to the region for the purpose of a holiday vacation [3].

Away from the beaches, resorts, and cruise ships, however, there lies a hidden underbelly of poverty in the Caribbean, and with this poverty, endemic neglected tropical diseases (NTDs) [4]. Shown in Table 1 are four Caribbean countries—Dominican Republic (DR), Guadeloupe, Haiti, and Jamaica—that exhibit an unusually high burden of NTDs. In addition, the island nations of Antigua and Barbuda, Barbados, Saint Lucia, and Trinidad and Tobago also stand out for their high NTD disease burdens. For example, of the Western Hemisphere’s 720,000 cases of lymphatic filariasis, a mosquito-transmitted disfiguring parasitic helminth infection caused by *Wuchereria bancrofti* that can result in elephantiasis, almost 90% of the cases occur in the Caribbean, including 560,000 cases in Haiti and 50,000 in DR [5]. Outside of Brazil, the second largest number of cases of the blood fluke infection, schistosomiasis (caused by *Schistosoma mansoni*), occur in the DR (258,000 cases) [6], especially in the eastern part of the island where the *Biomphalaria* snail vector is still present [7],[8]. Another 4,400 cases occur in Guadeloupe, and transmission still occurs in Saint Lucia and Antigua and Barbuda [6]. High prevalence of the three major intestinal helminth infections—ascariasis, trichuriasis, and hookworm infection—are also found throughout the poorest areas of the Caribbean, particularly in the DR, Haiti, and Jamaica, but also in Barbados and Trinidad and Tobago [9]. The existence of high rates of lymphatic filariasis, schistosomiasis, and hookworm infection in the region is made all the more poignant by an observation made recently in *PLoS Neglected Tropical Diseases* that these NTDs were most likely introduced into the Caribbean through the Atlantic slave trade [10], and even today such infections still occur almost exclusively among people living in poverty or people of African descent (P. Hotez, M. Bottazzi, C. Franco-Paredes, S. Ault, M. Roses Periago, unpublished data).

**Table 1:** Estimated Burden (Number of Cases) of Selected Neglected Tropical Diseases in the Dominican Republic, Guadeloupe, Haiti, and Jamaica.

Country (Population)	Ascariasis	Trichuriasis	Hookworm Infection	Dengue	Lymphatic Filariasis	Schistosomiasis
Dominican Republic (9.1 million)	456,643	628,962	94,775	5,540	50,000	258,000
Guadeloupe (0.4 million)	Not Determined	Not Determined	Not Determined	3,874	None	4,400
Haiti (8.1 million)	2,637,883	3,788,362	776,573	Not Determined	560,000	None
Jamaica (2.6 million)	559,852	1,205,124	81,427	52	None	None
Reference	[9]	[9]	[9]	[13]	[5]	[6]

In addition to the slavery-associated parasitic infections, there are other NTDs of great importance. Leprosy is still reported from Cuba, DR, Haiti, Jamaica, Saint Lucia, and Trinidad and Tobago [11], but because of the availability of multi-drug treatments, it is no longer considered a major public health threat to the region. However, dengue fever is now a serious problem, which is now common not only in the poorest Caribbean countries listed in Table 1, but also in Puerto Rico and other more developed areas [12]. The emergence of this mosquito-borne infection has been linked to an increase in flooding from hurricanes and other natural phenomena that may result from global warming [13]. The rat-borne zoonotic NTD leptospirosis has also emerged in Jamaica and elsewhere following flooding and hurricanes [14].

With the possible exceptions of dengue [15] and cutaneous larva migrans resulting from canine hookworm infection [16], most of the Caribbean’s NTDs are not considered significant threats to the health of American, Canadian, and European tourists. The real tragedy of these conditions is that despite the enormous amount of wealth infused into the Caribbean economy every year through tourism, very little if any trickles down to the poorest people in the region who suffer daily from chronic, debilitating, disfiguring, and stigmatizing NTDs. Equally tragic is the evidence base to support the feasibility of NTD elimination and at low cost. For instance, through a demonstration project conducted in Leogane, Haiti, annual mass drug administration (MDA) with diethylcarbamazine (DEC) and albendazole has resulted in the near elimination of lymphatic filariasis after five rounds [17]. DEC can be purchased for pennies per person, while albendazole is donated free-of-charge by GlaxoSmithKline [18]. Similarly, it is possible to eliminate schistosomiasis by MDA with the low-cost generic drug praziquantel and achieve dramatic reductions in the prevalence and intensity of the intestinal helminth infections ascariasis and trichuriasis through MDA with donated albendazole or mebendazole [19]. Given that industry is either donating the NTD drugs, or they are available as low-cost generic drugs, these last vestiges of American slavery could be controlled or eliminated for a ridiculously small amount. Indeed, if every American, Canadian, and European tourist over the course of a single year donated only US$1.00, the estimated $20 million generated should be sufficient to achieve the elimination of lymphatic filariasis and schistosomiasis from the Caribbean and achieve important reductions in morbidities from intestinal helminths [4]. Additional funds could also be set aside to develop new “antipoverty vaccines” to prevent hookworm infection, dengue, and other more intractable NTDs in the region [20].

The Global Network for NTDs has been established to mobilize resources for NTDs through MDA with donated or low-cost generic drugs [19]. I believe that there is an urgent need to identify an innovative financial mechanism for NTD control in the Caribbean through increased commitments by local governments or through external mechanisms such as a modest US$1.00 airline or cruise ship tax or a tax on tourist entry, or though more conventional donations from North American and European governments and private foundations. Such efforts would require careful coordination by organizations invested in the region, including the Caribbean Epidemiology Centre, the Pan American Health Organization, the United States Centers for Disease Control and Prevention, and the Global Network for NTDs, to ensure an equitable distribution of resources and universal access to essential medicines.

Given the impact of the NTDs on child development, pregnancy outcome, and worker productivity, ultimately, US$1.00 (less than the cost of a single piña colada!) per tourist in order to eliminate massive NTD health disparities in the Caribbean is a very reasonable and easy-to-digest financial mechanism, as well as a highly cost-effective means to lift the region’s poorest people out of poverty.
